# Updating singular value decomposition for modal analysis of slow-varying non-stationary vibration structures

**DOI:** 10.1177/10775463231214308

**Published:** 2023-11-24

**Authors:** Thi-Thuyet Bui, Viet-Hung Vu, Zhaoheng Liu, Marc Thomas

**Affiliations:** 1 14849 École de technologie supérieure, Montréal, QC, Canada; 2 85459 Royal Military College of Canada, Kingston, ON, Canada

**Keywords:** Autoregressive model, recursive least-squares, model order selection, model parameter identification, varying system, sliding window, singular value decomposition

## Abstract

This paper proposes a novel method for the modal analysis of slow-varying vibration structures based on vector autoregressive models. The basic idea of this method consists of using a short-time sliding window (STSW) to identify modal parameters for non-stationary vibrations. This method uses the recursive least-squares estimation for multivariable systems with the singular value decomposition (SVD) method to find the solutions within a segment of the data from each time window. Model identification is conducted by updating the SVD of the data matrix using the order and time from the previous computational window to monitor the modal parameters of a slow-varying system. Finally, this work was validated first by numerically simulating a system's gradual changes submitted to an exciting force and further by an experiment on a hydraulic turbine blade.

## 1. Introduction

System identification methods are generally divided into parametric and non-parametric methods (Petsounis and Fassois, 2001a and 2001b). Non-parametric methods are based on non-parameterized representations, which may be the impulse response function, the autocovariance function, and describing the signal’s power spectral density. Many studies have used the Hilbert-Huang transform (Shi et al., 2009), some focusing on the Cohen class of distribution (Lee et al., 2001; Meltzer and Ivanov, 2003; Roshan-Ghias et al., 2007), and others on the wavelet-based representation (Wang et al., 2013).

The parametric methods are the functional time-dependent autoregressive (TAR) series (Spiridonakos and Fassois, 2013), time-dependent autoregressive moving average (TARMA) series (Petsounis and Fassois, 2000), and specific functional subspaces. They have drawn much attention because of their broad application to many fields. Author Ma et al. (2018) presented the parametric output-only identification of time-varying structures using a kernel recursive extended least-squares TARMA approach. More specifically, the study used the TARMA model in kernel Hilbert space to track the time-varying dynamics. Other author Yang et al. (2015) proposed a moving kriging shape function modeling of vector TARMA models for modal identification and then validated the identification algorithm with a moving cantilever beam experiment. From another point of view, Li et al. (2019) presented a Bayesian estimation of operational modal parameters for linear time-varying mechanical systems based on the functional series vector TAR model. This built the analytical expression conjugate prior to the unknown parameters, the spanning AR coefficients, and showed the excellent performance of TAR models based on the Bayesian estimation for the time-varying vibration. Another method, employed by Spiridonakos and Fassois (2013), was to apply the stochastic functional series time-dependent autoregressive (FS-TAR) method in each state for effective fault diagnosis in inherently non-stationary structures, after which, the AR coefficients of the projection parameter vector are extracted and utilized as the characteristic quantity representing the structural state in each case. Based on parameterized representations of the time-dependent series models, parametric methods are advantageous in terms of their improved accuracy, resolution, and tracking of time-varying dynamics.

In real-life structural systems, the dynamic properties of these systems change under working conditions and are known as non-stationary systems (Chen et al., 2020). Typical structural systems include traffic-excited bridges, earthquake-excited structures, surfaces of any kind, sea vehicles, robotic devices, and rotating machinery (Au et al., 2004; Verboven et al., 2004; Vu et al., 2016). The extraction of modal parameters for non-stationary systems is more complex than for stationary systems whose dynamic properties remain constant over time.

The methods for non-stationary system identification may be classified as fast and slow non-stationary (fast and slow time-varying). In the fast non-stationary methods, the parameters are explicit functions of time. The slow non-stationary methods, on the other hand, are based on conventional stationary frequencies or time domain system identification and signal segmentation techniques. There are several difficulties in developing algorithms for identifying fast non-stationary systems. The presence of time-dependent coefficients results in more computational complexity and matrix singularity. In addition, it is very challenging to choose the functions for time-varying parameters in the methods for non-stationary vibration systems.

Therefore, the slow non-stationary methods have drawn much attention thanks to their potential application in various practical systems. Under the assumptions of short time-invariance and the theories of “time‐freezing” (Zadeh, 1950), the time-varying systems can be regarded as time-invariant systems over short periods. The vibration response signals of slow-varying systems are entirely obtained simultaneously, but they can be received by continuous sampling over time. Many researchers have developed methods to extract modal parameters for the slow-varying non-stationary vibration systems (Verboven et al., 2004; Cheng et al., 2020; Zong et al., 2020). Although many problems have been solved, the methods for slow-varying non-stationary vibration remain limited as computational complexity and matrix singularity. Hence, developing the algorithms for slow-varying non-stationary structures to overcome these disadvantages is an open problem. Furthermore, updating the computational model to track and monitor the modal parameters can be developed in conjunction with the identification method to provide a more efficient online modal analysis technique.

The time-varying autoregressive model (TVAR) is similar to the conventional autoregressive (AR) model. However, TVAR has more time-varying coefficients that could lead to certain disadvantages, such as computational complexity and matrix singularity for the identification. In addition, the time-varying system’s ambient excitation is usually difficult to measure under operating conditions. A powerful technique to mitigate these disadvantages is the locally stationary method based on the conventional stationary frequency domain or time domain system identification and signal segmentation technics. Many endeavors have been made in this direction. Author Ma and Ding (2019) assumed that the system parameters vary linearly with time in each window. A linear function describes the temporal variation of the parameter in the shifting window. Hence, the time-varying parameters are identified in the different time windows. In another identification approach, the short-time autoregressive (STAR) modeling was used for the operational modal analysis of a non-stationary mechanical system (Vu et al., 2011a). Based on the stationary state of each data segment, the modal parameter variations are monitored by autoregressive models for the emerging steel plate.

With respect to the mathematical component, the singular value decomposition method is a widely used technique to decompose a matrix into several component matrices (Gandhi and Rajgor, 2017; Stewart, 2006). It has been used in system identification (Brincker et al., 2001; Shen and Wai, 2021; Sun et al., 2021) to monitor the modal parameters for both cases: stationary and non-stationary vibrations. Other author Jiang et al. (2021) have presented a method of damage detection using singular value decomposition (SVD) for beam structures. SVD is applied to decompose the trajectory matrix of the attractor reconstructed from shape data to localize the damage to detect the defects for beam-like systems, simplifying the measurement method and reducing testing work. Another technique, found in Lobos et al. (2001), is to use singular value decomposition (SVD) to estimate the harmonics in signals in the presence of high noise. The method was developed to locate the frequencies in closely spaced sinusoidal signals. The study also presented the superiority of SVD with the standard FFT technique for signals buried in the noise. It concluded that the SVD method is especially suitable for offline analysis of recorded waveforms.

The singular value decomposition of a matrix is a valuable and important method used in the least-squares fitting of data. Many applications, for instance, signal processing, mechanical engineering, or statistics, employ SVD. In many cases, the computing procedure of SVD is repeated. This repetition could lead to high computational costs. Some authors have shared that updating the SVD is a further development that overcomes this drawback. Such an update algorithm, due to Businger, is described in Bunch and Nielsen (1978). It is reliable and efficient for a matrix with SVD and is applied when adding or deleting a row or column. In Brand (2006), author recently developed a fast algorithm to update a few dominant singular values of an augmented matrix used to perform background elimination in multiple analysis systems. A thin SVD is calculated through a matrix’s column updates and downdates.

This paper proposes a new algorithm for the online monitoring of slow-varying modal parameters in vibrating structures subjected to unknown excitation. The proposed method applies a vector autoregressive model (VAR) in a short-time sliding window (STSW) on measured signals. The model parameters are determined and updated through the order and time from the previous computational window. The recursive least-squares estimation for multivariable systems is used to find the solutions by the singular value decomposition (SVD). This work aims to avoid the computational complexity of identifying and monitoring modal parameter variations for non-stationary vibrations. In Bui et al. (2022), the Schur complement was used to update the parameters of the VAR model and monitor the varying modal parameters for a submerged plate. In terms of computational time, the Schur method is fast because it solves the standard equations of the least-squares. However, the main obstacle to vibration signal analysis is that the collected non-stationary signals are usually mixed with heavy noise caused by variable operating or environmental conditions. As a result, the rank deficient in least-squares estimation must be coped with to overcome this problem. This problem can be better handled by the generalized Schur complement (Ando, 1979). However, in this paper we are using the singular value decomposition method, which is a highly reliable, computationally stable mathematical tool that could obtain more accurate results and help to resolve these problems.

This study is structured as follows. Section 2 briefly introduces the vector autoregressive models and singular value decomposition. The updated approximation for the singular value decomposition of the matrix will be discussed in Section 3. Section 4 presents the proposed method for updating the modal parameters of the VAR model. The identification of the mechanical and operational systems will be presented in Section 5. The conclusion is summarized in the final Section.

## 2. Vector autoregressive models and singular value decomposition

Considering the general time-invariant recursive process for signal $y\left[t\right]\in R^{1\times n}$ , referred to as a multivariate autoregressive model at p , dimension n and sampling period $T_{s}$ , that is given by the following equation (Vu et al., 2011b):

(1) $$y\left[t\right]+\overset{p}{\underset{i=1}{\sum }}A_{i}y\left[t-i\right]=e\left[t\right]$$

where t designates the normalized discrete time, $e\;\left[t\right]\in R^{1\times n}$ a residual vector with zero means, and $A_{i}\in R^{n\times 1}$ the AR parameter matrix. Equation (1) is rewritten into the following linear regression form:

(2) $$y\left[t\right]=z\left[t\right]P_{np\times n}+e\left[t\right]$$

where $z\left[t\right]={\left(y\left[t-1\right],y\left[t-2\right],\ldots ,y\left[t-p\right]\right)}^{T}\in R^{1\times np}$ is the corresponding regression vector, and

$P_{np\times n}=\left[-A_{1}-A_{2}\;\ldots \;-A_{p}\right]$ is the AR parameter matrix.

A least-squares estimation can be applied if the data are assumed to be measured in a white-noise environment. Considering N successive vectors of the output responses from y [t] to y [t+N−1] , the modal parameters matrix $P_{np\times n}$ can be found in the least-squares method by minimizing the summed squared error between the left and right-hand sides of the equation. The objective function to be minimized may be expressed in the norm-2 vector notation form as follows (Lobos et al., 2001):

(3) $$E=\frac{1}{2}{\left\|K_{N\times np}P_{np\times n}-Y{\left[t\right]}_{N\times n}\right\|}_{2}^{2}$$

where

(4) $$Y{\left[t\right]}_{N\times n}=\left[\begin{array}{l} y\left[t\right]\\ y\left[t+1\right]\\ \ldots \\ y\left[t+N-1\right] \end{array}\right],K_{N\times np}=\left[\begin{array}{l} z\left[t\right]\\ z\left[t+1\right]\\ \ldots \\ z\left[t+N-1\right] \end{array}\right]$$

The singular value decomposition of the $K_{N\times np}$ matrix is used to compute the solution of the least-squares method. There are orthogonal matrices $U_{N\times np}$ , $V_{np\times np}$ , and a diagonal matrix $D_{np\times np}$ such that $K_{N\times np}=\text{UD}V^{T}$ . Here, $U_{N\times np}$ and $V_{np\times np}$ are the left singular vectors and the suitable right singular vectors of $K_{N\times np}$ , respectively, and the diagonal entries $D=diag\left(d_{1},d_{2},\ldots ,d_{np}\right)$ are the singular values of $K_{N\times np}$ . The model parameters of the AR model are estimated as follows (Lobos et al., 2001):

(5) $$P_{np\times n}=VD^{-1}U^{T}Y{\left[t\right]}_{N\times n}$$

A state matrix of the AR model at order p is constructed from the AR coefficient matrix, that is,

(6) $$A_{np\times np}=\left[\begin{array}{llllll} -A_{1} & -A_{2} & -A_{3} & \ldots & \ldots & -A_{p}\\ I & 0 & 0 & \ldots & \ldots & 0\\ 0 & I & 0 & \ldots & \ldots & 0\\ \ldots & \ldots & \ldots & \ldots & \ldots & \ldots \\ 0 & 0 & 0 & \ldots & I & 0 \end{array}\right]$$

where $I\in R^{n\times n}$ is the identity matrix. The eigenvalue decomposition of the state matrix to determine modal parameters of a mechanic system is presented as:

(7) $$A_{np\times np}=L\Lambda L^{-1}=L\left|\begin{array}{llll} g_{1} & 0 & 0 & 0\\ 0 & g_{2} & 0 & 0\\ 0 & 0 & \ddots & \vdots \\ 0 & 0 & \ldots & g_{np} \end{array}\right|L^{-1}$$

where $g_{i},i=1,2,\ldots ,np$ are discrete eigenvalues and $L\in R^{np\times np}$ are eigenvectors of the state matrix. Considering that, each complex eigenvalue $g_{i}$ of the discrete system corresponds to one natural frequency of the mechanical system, then: $\lambda_{i}=\frac{\ln\left(g_{i}\right)}{T_{s}}$ . Therefore, the natural frequencies $f_{i}$ and $\xi_{i}$ damping ratio are computed from complex conjugate pairs $\lambda_{i}$ as follows:

(8) $$f_{i}=\frac{\sqrt{{\text{Re}}^{2}\left(\lambda_{i}\right)+{\text{Im}}^{2}\left(\lambda_{i}\right)}}{2\pi },\xi_{i}=-\frac{\text{Re}\left(\lambda_{i}\right)}{2\pi f_{i}}$$

## 3. Updating the singular value decomposition of a matrix

In many least-squares and signal processing applications, one updates a matrix $K_{N\times p}$ by appending or deleting a row or a column. After each update or downdate, the computing process of the SVD must be repeated for the resulting matrix. This section presents the updating formulation SVD for the matrix when appending and deleting a row or a column (Bunch and Nielsen, 1978).

Consider the singular value decomposition of a given matrix $K_{N\times p}$ as:

(9) $$K_{N\times p}=UDV^{T}$$

where $U\in R^{N\times p}$ and $V\in R^{p\times p}$ are orthogonal, and $D\in R^{p\times p}$ is zero except on the main diagonal $D=diag\left(d_{1},d_{2},\ldots ,d_{p}\right)$ .

### 3.1. Updating the SVD of a matrix when appending a row

Define a new matrix ${\tilde{K}}_{\left(N+1\right)\times p}$ that is based on the given matrix $K_{N\times p}$ when appending a row $a^{T}$ as follows:

(10) $${\tilde{K}}_{\left(N+1\right)\times p}=\left(\begin{array}{l} K_{N\times p}\\ a^{T} \end{array}\right)=\tilde{U}\tilde{D}{\tilde{V}}^{T}$$

where $\tilde{U}\in R^{\left(N+1\right)\times p},\tilde{V}\in R^{p\times p},\tilde{D}\in R^{p\times p},\tilde{D}=diag\left({\tilde{d}}_{1},{\tilde{d}}_{2},\ldots ,{\tilde{d}}_{p}\right)$ .

One can compute $\tilde{U},\tilde{D},\tilde{V}$ matrices of ${\tilde{K}}_{\left(N+1\right)\times p}$ by using the provided information of the matrices U,D,V of $K_{N\times p}$ .

With $z=V^{T}a={\left[z_{1}\;z_{2}\;\ldots \;z_{p}\right]}^{T}$ , a factorized representation, is defined by:

(11) $${\tilde{K}}_{\left(N+1\right)\times p}^{T}{\tilde{K}}_{\left(N+1\right)\times p}=K_{N\times p}^{T}K_{N\times p}+aa^{T}=U\left(D^{2}+zz^{T}\right)V^{T}$$

From equation (11), the singular values of ${\tilde{K}}_{\left(N+1\right)\times p}$ are computed by the eigen decomposition of the matrix $D^{2}+zz^{T}$ . The SVD of $D^{2}+zz^{T}$ is expressed by: $D^{2}+zz^{T}=Q\Omega^{2}Q^{T}$ . Here, $Q\in R^{p\times p}$ is the orthogonal and $\tilde{D}=\Omega \in R^{p\times p}$ .

The singular values of the new matrix are updated through the singular values of the matrix $K_{N\times p}$ . The singular values of the new matrix can be updated as by the method developed in Bunch and Nielsen (1978) as follows:

(12) $${\tilde{d}}_{i}=d_{i}+\mu_{i},1\leq i\leq p$$

where $\mu_{i}$ satisfy the secular equation, more details on $\mu_{i}$ can be found in Bunch and Nielsen (1978):

(13) $$1+\overset{p}{\underset{i=1}{\sum }}\frac{z_{i}^{2}}{\left(d_{i}+d_{j}+\mu \right)\left(d_{j}-d_{i}-\mu \right)}=0,\;1\leq j\leq p$$

Instead of computing the singular values directly of the matrix ${\tilde{K}}_{\left(N+1\right)\times p}$ , one can update them through the secular equation (13). Once the singular values of the new matrix have been updated, the right singular vector $\tilde{V}=\left[{\tilde{v}}_{1}\;{\tilde{v}}_{2}\;\ldots \;{\tilde{v}}_{p}\right]$ is obtained by:

(14) $${\tilde{v}}_{i}={\frac{VT_{i}^{-1}z_{1}}{\left\|T_{i}^{-1}z_{1}\right\|}}_{2},T_{i}=D^{2}-{\tilde{d}}_{i}I,z_{1}=\frac{z}{{\left\|a\right\|}_{2}}$$

The updated left singular vectors $\tilde{U}=\left[{\tilde{u}}_{1}\;{\tilde{u}}_{2}\;\ldots \;{\tilde{u}}_{p}\right]$ are related to the updated right singular vectors as:

(15) $${\tilde{u}}_{i}=\frac{1}{{\tilde{d}}_{i}}\left(\begin{array}{l} K_{N\times p}\\ a^{T} \end{array}\right){\tilde{v}}_{i},1\leq i\leq p$$

Obviously, the need to repeat SVD on equation (10)’s matrix is avoided by using the updated formulas from the four equations (12)–(15), which further reduces the computational complexity of computing SVD. Thus, solving the least-squares problem by SVD is beneficial for the identification process.

### 3.2. Updating the SVD of a matrix when deleting a row

A new matrix based on the given matrix $K_{N\times p}$ when deleting a row $a^{T}$ is defined as equation (16):

(16) $$K_{N\times p}=\left(\begin{array}{l} K_{\left(N-1\right)\times p}^{\%}\\ a^{T} \end{array}\right)=UDV^{T}$$

where ${\tilde{K}}_{\left(N-1\right)\times p}=\tilde{U}\tilde{V}\tilde{D},\tilde{U}\in R^{\left(N-1\right)\times p},\tilde{V}\in R^{p\times p},\tilde{D}\in R^{p\times p},\tilde{D}=diag\left({\tilde{d}}_{1},{\tilde{d}}_{2},\ldots ,{\tilde{d}}_{p}\right)$

Equation (16) implies that ${\tilde{K}}_{\left(N-1\right)\times p}^{T}{\tilde{K}}_{\left(N-1\right)\times p}=K_{N\times p}^{T}K_{N\times p}-aa^{T}=U\left(D^{2}-zz^{T}\right)V$ , where $=V^{T}a={\left[z_{1}\;z_{2}\;\ldots \;z_{p}\right]}^{T}$ .

Thus, the singular values of ${\tilde{K}}_{\left(N-1\right)\times p}$ can be found by computing the eigen decomposition of $D^{2}-zz^{T}=Q\Omega^{2}Q^{T}$ , where $Q\in R^{p\times p}$ is the orthogonal matrix, and $\tilde{D}=\Omega \in R^{p\times p}$ is a non-negative and diagonal matrix.

The singular values decomposition of the matrix ${\tilde{K}}_{\left(N-1\right)\times p}$ is defined through the SVD of the matrix $K_{N\times p}$ as follows (Bunch and Nielsen, 1978):

(17) $${\tilde{d}}_{i}=d_{i}+\mu_{i},1\leq i\leq p$$

where $\mu_{i}$ satisfy the secular equation:

(18) $$-1+\overset{p}{\underset{i=1}{\sum }}\frac{z_{i}^{2}}{\left(d_{i}+d_{j}+\mu \right)\left(d_{j}-d_{i}-\mu \right)}=0,\;1\leq j\leq p$$

The updated right singular vectors $\tilde{V}=\left[{\tilde{v}}_{1}\;{\tilde{v}}_{2}\;\ldots \;{\tilde{v}}_{p}\right]$ and the updated left singular vectors $\tilde{U}=\left[{\tilde{u}}_{1}\;{\tilde{u}}_{2}\;\ldots \;{\tilde{u}}_{p}\right]$ are estimated as forms:

(19) $${\tilde{v}}_{i}={\frac{VT_{i}^{-1}z}{\left\|T_{i}^{-1}z\right\|}}_{2},T_{i}=D^{2}-{\tilde{d}}_{i}I,1\leq i\leq p$$

(20) $${\tilde{u}}_{i}=\frac{1}{{\tilde{d}}_{i}}{\tilde{K}}_{\left(N-1\right)\times p}{\tilde{v}}_{i},1\leq i\leq p$$

Thus, the set of four equations (17)–(20) is an effective procedure for updating the SVD of the matrix when deleting a row. The aim of this technique is to reduce the computational complexity when computing the SVD of a matrix.

### 3.3. Updating the SVD of a matrix when appending a column

In this case, a matrix based on the given matrix $K_{N\times p}$ when adding a column is defined as:

(21) $${\tilde{K}}_{N\times \left(p+1\right)}=\left(K_{N\times p}\;b\right)=\tilde{U}\tilde{D}{\tilde{V}}^{T}$$

where $\tilde{U}\in R^{N\times \left(p+1\right)},\tilde{V}\in R^{\left(p+1\right)\times \left(p+1\right)},\tilde{D}\in R^{\left(p+1\right)\times \left(p+1\right)},\tilde{D}=diag\left({\tilde{d}}_{1},{\tilde{d}}_{2},\ldots ,{\tilde{d}}_{p+1}\right)$

Letting $z^{\prime }=U^{T}b={\left[z_{1}^{\prime }\;z_{2}^{\prime }\;\ldots z^{\prime }\right]}^{T},\alpha =\frac{1}{{\left\|b\right\|}_{2}}$ , the factorized representation can be rewritten as:

(22) $${\tilde{K}}_{N\times \left(p+1\right)}^{T}{\tilde{K}}_{N\times \left(p+1\right)}=K_{N\times p}^{T}K_{N\times p}+bb^{T}=U\left(D^{2}+z^{\prime }{z^{\prime }}^{T}\right)V^{T}$$

The eigen decomposition of $D^{2}+z^{\prime }{z^{\prime }}^{T}$ can be written as $Q\Omega^{2}Q^{T}$ , where $Q\in R^{\left(p+1\right)\times \left(p+1\right)}$ , $\tilde{D}=\Omega \in R^{\left(p+1\right)\times \left(p+1\right)}$ .

The singular values of the matrix ${\tilde{K}}_{N\times \left(p+1\right)}$ are updated via the eigen decomposition of $D^{2}+z^{\prime }{z^{\prime }}^{T}$ as follows (Bunch and Nielsen, 1978):

(23) $${\tilde{d}}_{i}=d_{i}+\mu_{i},1\leq i\leq p+1$$

where $\mu_{i}$ satisfy the secular equation:

(24) $$1+\frac{1}{\alpha^{2}}\overset{p}{\underset{i=1}{\sum }}\frac{{z^{\prime }}_{i}^{2}}{\left(d_{i}+d_{j}+\mu \right)\left(d_{j}-d_{i}-\mu \right)}-\frac{{\left(1-{\left\|z^{\prime }\right\|}_{2}^{2}\right)}^{2}}{\alpha^{2}\mu }=0,\;1\leq j\leq p+1$$

The updated right singular vector $\tilde{V}=\left[{\tilde{v}}_{1}\;{\tilde{v}}_{2}\;\ldots \;{\tilde{v}}_{p+1}\right]$ is determined through the left singular vector $V\in R^{p\times p}$ and the regular values $\tilde{D}\in R^{\left(p+1\right)\times \left(p+1\right)}$ form:

(25) $${\tilde{v}}_{i}=\eta_{i}\left[\begin{array}{l} VT_{i}^{-1}Dz^{\prime }\\ -1 \end{array}\right],T_{i}=D^{2}-{\tilde{d}}_{i}I,\eta_{i}={\left\|\left[\begin{array}{l} VT_{i}^{-1}Dz^{\prime }\\ -1 \end{array}\right]\right\|}_{2}^{-1}$$

The updated left singular vectors $\tilde{U}=\left[{\tilde{u}}_{1}\;{\tilde{u}}_{2}\;\ldots \;{\tilde{u}}_{p+1}\right]$ are updated via the right singular vectors and the given matrix by:

(26) $${\tilde{u}}_{i}=\frac{1}{{\tilde{d}}_{i}}\left(K_{N\times p}\;b\right){\tilde{v}}_{i},1\leq i\leq \left(p+1\right)$$

Instead of repeating the computing SVD of the matrix, the SVD updating procedure of the matrix when appending a column is presented in the set of four equations (23)–(26).

## 4. Modal analysis of non-stationary vibrations

As presented in the previous section, many studies have used the matrix's singular value decomposition (SVD) in system identifications. Updating the SVD of the matrices by appending or deleting a column and a row has been presented in many algorithms. This section develops a formula for updating model parameters for AR models by updating the SVD of a data matrix through the identification procedure with respect to time and model order. The proposed process for the modal analysis is also presented in this section.

### 4.1. Updating in model order for AR model parameters

Consider that, the data matrix $K_{N\times np}$ and the output vector $Y{\left[t\right]}_{N\times n}$ of the AR at order p are formed from N successive samples by:

(27) $$Y{\left[t\right]}_{N\times n}=\left[\begin{array}{l} y\left[t\right]\\ y\left[t+1\right]\\ \ldots \\ y\left[t+N-1\right] \end{array}\right],K_{N\times np}^{\left(p\right)}=\left[\begin{array}{l} z\left[t\right]\\ z\left[t+1\right]\\ \ldots \\ z\left[t+N-1\right] \end{array}\right]$$

The goal of the model identification is to determine all the model parameters. Based on equation (5), solving the least-squares problem for the model parameters of AR models by SVD is defined by:

(28) $$P_{np\times n}=V_{\left(p\right)}D_{\left(p\right)}^{-1}U_{\left(p\right)}^{T}Y{\left[t\right]}_{N\times n}$$

where $K_{N\times np}^{\left(p\right)}=U_{\left(p\right)}D_{\left(p\right)}V_{\left(p\right)}^{T}$ with $U_{\left(p\right)}\in R^{N\times np},V_{\left(p\right)}\in R^{np\times np}$ are orthogonal matrices, and $D_{\left(p\right)}\in R^{np\times np}$ is a square diagonal matrix.

The data matrix at order p+1 can be built by appending a data sub-matrix into the data matrix $K_{N\times np}^{\left(p\right)}$ as follows:

$$K_{N\times n\left(p+1\right)}^{\left(p+1\right)}=\left(K_{N\times np}^{\left(p\right)}K_{N\times n}^{\prime }\right),\text{where}K_{N\times n}^{\prime }=\left[\begin{array}{l} y\left[t-\left(p+1\right)\right]\\ y\left[t+1-\left(p+1\right)\right]\\ \ldots \\ y\left[t+N-1-\left(p+1\right)\right] \end{array}\right]$$

**Figure 1.**
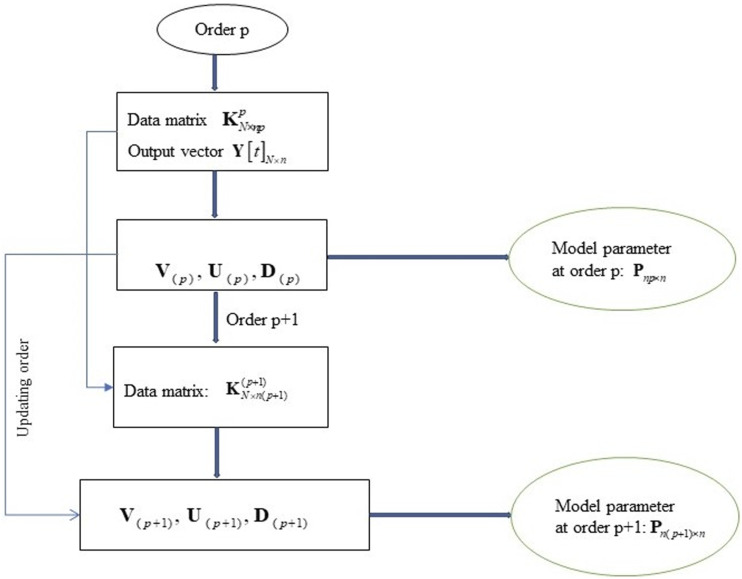
Flow diagram of updating order for model parameters.

The model parameter estimation of AR models at order p+1 is obtained by updating the SVD of the data matrix. The flow diagram of updating order for model parameters is presented in Figure 1. The proposed algorithm is summarized as follows:

Input: $K_{N\times np}^{\left(p\right)},K_{N\times n}^{\prime },Y{\left[t\right]}_{N\times n},U_{\left(p\right)},D_{\left(p\right)},V_{\left(p\right)},z^{\prime }=U^{T}K_{N\times n}^{\prime }={\left[z_{1}^{\prime }\;z_{2}^{\prime }\;\ldots \;z_{np}^{\prime }\right]}^{T},\alpha ={\left\|K_{N\times n}^{\prime }\right\|}_{2}^{-1}$

Step 1. Computation of the model parameters at order p .

Using the SVD in equation (9) of the data matrix to estimate the model parameters at order p in equation (28).

(29) $$P_{np\times n}=V_{\left(p\right)}D_{\left(p\right)}^{-1}U_{\left(p\right)}^{T}Y{\left[t\right]}_{N\times n}$$

Step 2. Update of the singular values of the data matrix at order p+1 .

Solving the secular equation in equation (24) to calculate the singular values of $D_{\left(p+1\right)}$ via $z^{\prime },D_{\left(p\right)}$ at order p .

Step 3. Update of the right vector $V_{\left(p+1\right)}$ of the data matrix at order (p+1) .

Based on $V_{\left(p\right)},z^{\prime },D_{\left(p\right)},D_{\left(p+1\right)}$ , the right vectors $V_{\left(p+1\right)}$ at order (p+1) are updated in equation (25).

Step 4. Update of the left vectors $U_{\left(p+1\right)}$ of the data matrix at order p+1 .

Once the singular values and the right vectors at order p+1 are obtained, the left vectors $U_{\left(p+1\right)}$ are updated through $V_{\left(p\right)},z^{\prime },D_{\left(p\right)},D_{\left(p+1\right)},K_{N\times np}^{\left(p\right)},K_{N\times n}^{\prime }$ in equation (26).

Step 5. Computation of the model parameters of AR models at order (p+1) .

Because the singular values, the left and right vectors at order p+1 , are calculated via $U_{\left(p\right)},D_{\left(p\right)},V_{\left(p\right)}^{T}$ at order p , the solution of the least-square problem $P_{n\left(p+1\right)\times n}=V_{\left(p+1\right)}D_{\left(p+1\right)}^{-1}U_{\left(p+1\right)}^{T}Y{\left[t\right]}_{N\times n}$ can be directly updated.

The above algorithm identifies the model parameters at higher model orders through the SVD of the data matrix at the previous order. The model parameters are defined with low computational complexity and computational time effectiveness. This technique is preferable to the repetitive approach of equation (28) for each order value.

### 4.2. Updating in time for AR model parameters

Observation matrix $K{\left[k\right]}_{N\times np}$ and output vector $Y{\left[k\right]}_{N\times n}$ at time t=k of the AR at order p are extracted from the measured portion of the global response as follows:

(30) $$K{\left[k\right]}_{N\times np}=\left[\begin{array}{l} z\left[k\right]\\ z\left[k+1\right]\\ \ldots \\ z\left[k+N-1\right] \end{array}\right],Y{\left[k\right]}_{N\times n}=\left[\begin{array}{l} y\left[k\right]\\ y\left[k+1\right]\\ \ldots \\ y\left[k+N-1\right] \end{array}\right]$$

Based on equation (5), solving the least-squares problem for the model parameters of AR models by SVD is defined as:

(31) $$P{\left[k\right]}_{np\times n}=V\left[k\right]D{\left[k\right]}^{-1}U{\left[k\right]}^{T}Y{\left[k\right]}_{N\times n}$$

where $K{\left[k\right]}_{N\times np}=U\left[k\right]D\left[k\right]V{\left[k\right]}^{T}$ . At time t=k+m , the data matrix of AR models can be represented by:

(32) $$K{\left[k+m\right]}_{N\times np}=\left[\begin{array}{l} z\left[k+m\right]\\ \ldots \\ z\left[k+N+1\right]\\ \ldots \\ z\left[k+m+N-1\right] \end{array}\right],Y{\left[k+m\right]}_{N\times n}=\left[\begin{array}{l} y\left[k+m\right]\\ y\left[k+m+1\right]\\ \ldots \\ y\left[k+m+N-1\right] \end{array}\right]$$

From the data matrices of the model at time t=k and t=k+m , one can rewrite equations (30) and (32), respectively, as:

(33) $$K{\left[k\right]}_{N\times np}=\left[\begin{array}{l} z\left[k\right]\\ z\left[k+1\right]\\ \ldots \\ z\left[k+N-1\right] \end{array}\right]=\left[\begin{array}{l} R_{1_{m\times np}}\\ R_{\left(N-m\right)\times np} \end{array}\right],K{\left[k+m\right]}_{N\times np}=\left[\begin{array}{l} z\left[k+m\right]\\ \ldots \\ z\left[k+N+1\right]\\ \ldots \\ z\left[k+m+N-1\right] \end{array}\right]=\left[\begin{array}{l} R_{\left(N-m\right)\times np}\\ R_{2_{m\times np}} \end{array}\right]$$

where

(34) $$R_{1_{m\times np}}=\left[\begin{array}{l} z\left[k\right]\\ \ldots \\ z\left[k+m-1\right] \end{array}\right],R_{\left(N-m\right)\times np}=\left[\begin{array}{l} z\left[k+m\right]\\ \ldots \\ z\left[k+N-1\right] \end{array}\right],R_{2_{m\times np}}=\left[\begin{array}{l} z\left[k+N\right]\\ \ldots \\ z\left[k+m+N-1\right] \end{array}\right]$$

**Figure 2.**
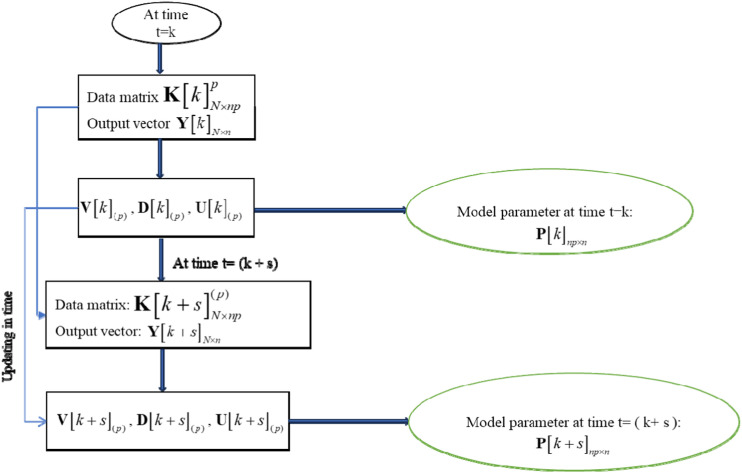
Flow diagram of updating in time for model parameters.

Instead of the iterative solution procedure required to calculate the model parameters at time t=k+m , the model parameters of the AR model at time t=k+m can be obtained by updating the SVD of the matrix. Figure 2 presents the flow diagram of updating in time for model parameters. The algorithm is implemented in five steps:

Input: $K{\left[k\right]}_{N\times np},Y{\left[k\right]}_{N\times n},{R_{1}}_{m\times np},{R_{2}}_{m\times np},R_{\left(N-m\right)\times np},Y{\left[k+m\right]}_{N\times n},U\left[k\right],D\left[k\right],V\left[k\right]$

Step 1Computation the model parameters at time t=k .

The model parameters of the AR model at time t=k are calculated using equation (31).

Step 2Update of the singular values of the data matrix at time t=k+m .

The singular values of the data matrix $K{\left[k+m\right]}_{N\times np}$ are identified by solving the secular equation in equations (13) and (18).

Step 3Update of the right vectors V[k+m] of the data matrix at time t=k+m .

Once, the singular values of the data matrix $K{\left[k+m\right]}_{N\times np}$ at time t=k+m are obtained in Step 2. The right vector are identified by equations (19) and (14) through ${R_{1}}_{m\times np},{R_{2}}_{m\times np},R_{\left(N-m\right)\times np},U\left[k\right],D\left[k\right],V\left[k\right]$ .

Step 4Update of the left vectors U[k+m] of the data matrix at time t=k+m .

The left vectors U[k+m] are computed using equations (15) and (20) through

$${R_{1}}_{m\times np},{R_{2}}_{m\times np},R_{\left(N-m\right)\times np},V\left[k+m\right],D\left[k\right],D\left[k+m\right]$$

Step 5Computation of the model parameters of the AR models at time t=k+m .

The AR model’s model parameters are obtained by using the updated singular values, right vectors, and left vectors in steps 2, 3, and 4 as: $P{\left[k+m\right]}_{np\times n}=V\left[k+m\right]D{\left[k+m\right]}^{-1}U{\left[k+m\right]}^{T}Y{\left[k+m\right]}_{N\times n}$ .

From this algorithm, the parameters of models t=k+m are updated through the SVD of the data matrix at the time t=k .

## 5. Results and discussion

In this section, the proposed method will be applied to a lumped-mass dynamic system and to a hydraulic turbine blade experimental setup to extract the modal parameters and monitor the systems.

### 5.1. Lumped-mass mechanical model

**Figure 3.**
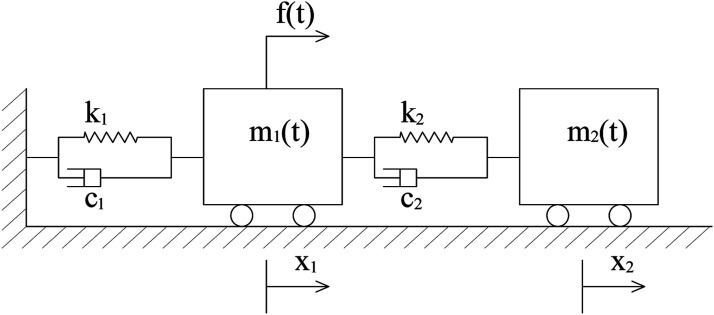
Schematic model of a two-degree-of-freedom (2-DOF) time-varying mechanical system illustrating its key components and the dynamic interactions between them.

A numerical simulation was carried out to produce numerical system input-output data. A mechanical model of the simulated system used for this study is shown in Figure 3. The dynamical model of the system was derived in equation (35).

The motion equation of the system can be derived from either a Newton or Lagrange formulation, as follows:

(35) $$\left[\begin{array}{l} m_{1}\left(t\right)\;0\\ 0\;m_{2}\left(t\right) \end{array}\right]\left\{\begin{array}{l} {\ddot{x}}_{1}\left(t\right)\\ {\ddot{x}}_{2}\left(t\right) \end{array}\right\}+\left[\begin{array}{l} c_{1}+c_{2}+{\dot{m}}_{1}\left(t\right)\;-c_{2}\\ -c_{2}\;c_{2}+{\dot{m}}_{2}\left(t\right) \end{array}\right]\left\{\begin{array}{l} {\dot{x}}_{1}\left(t\right)\\ {\dot{x}}_{2}\left(t\right) \end{array}\right\}+\left[\begin{array}{l} k_{1}+k_{2}\;-k_{2}\\ -k_{2}\;k_{2} \end{array}\right]\left\{\begin{array}{l} x_{1}\left(t\right)\\ x_{2}\left(t\right) \end{array}\right\}=\left\{\begin{array}{l} f\left(t\right)\\ 0 \end{array}\right\}$$

where ${\dot{m}}_{1}\left(t\right)=\frac{dm_{1}\left(t\right)}{dt},{\dot{m}}_{2}\left(t\right)=\frac{dm_{2}\left(t\right)}{dt}$ .

Because the variation of the masses is small in this example, the derivative of the masses with respect to time is negligible in the damping matrix. Therefore, equation (35) can be rewritten as follows:

(36) $$\left[\begin{array}{l} m_{1}\left(t\right)\;0\\ 0\;m_{2}\left(t\right) \end{array}\right]\left\{\begin{array}{l} {\ddot{x}}_{1}\left(t\right)\\ {\ddot{x}}_{2}\left(t\right) \end{array}\right\}+\left[\begin{array}{l} c_{1}+c_{2}\;-c_{2}\\ -c_{2}\;c_{2} \end{array}\right]\left\{\begin{array}{l} {\dot{x}}_{1}\left(t\right)\\ {\dot{x}}_{2}\left(t\right) \end{array}\right\}+\left[\begin{array}{l} k_{1}+k_{2}\;-k_{2}\\ -k_{2}\;k_{2} \end{array}\right]\left\{\begin{array}{l} x_{1}\left(t\right)\\ x_{2}\left(t\right) \end{array}\right\}=\left\{\begin{array}{l} f\left(t\right)\\ 0 \end{array}\right\}$$

**Figure 4.**
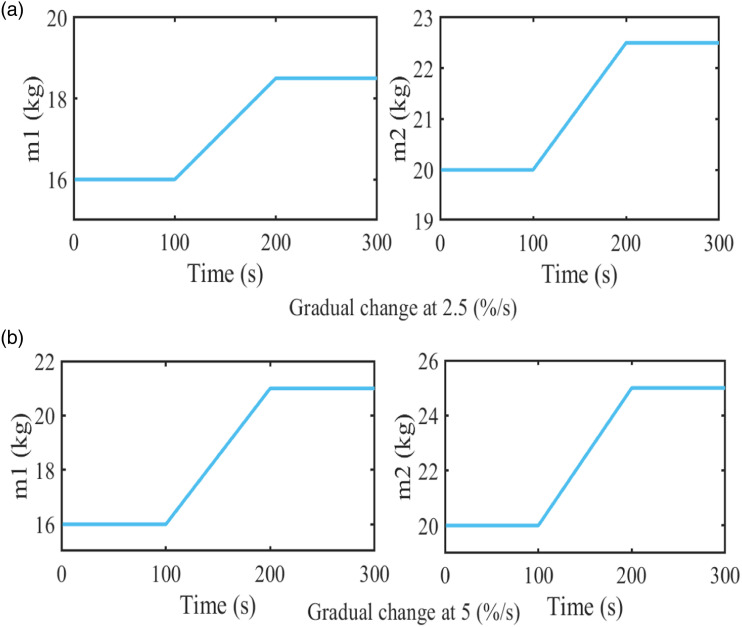
Mass changing function.

An excitation signal with a white-noise shape is applied in this sub-section with the different rate change of system’s masses. Figure 4 shows the gradual changing masses for two different rates. The numerical values of the system parameters are given as follows:

$$c_{1}=10\left(Ns/m\right),c_{2}=20\left(Ns/m\right),k_{1}=10000\;\left(N/m\right),k_{2}=22000\;\left(N/m\right)$$

**Figure 5.**
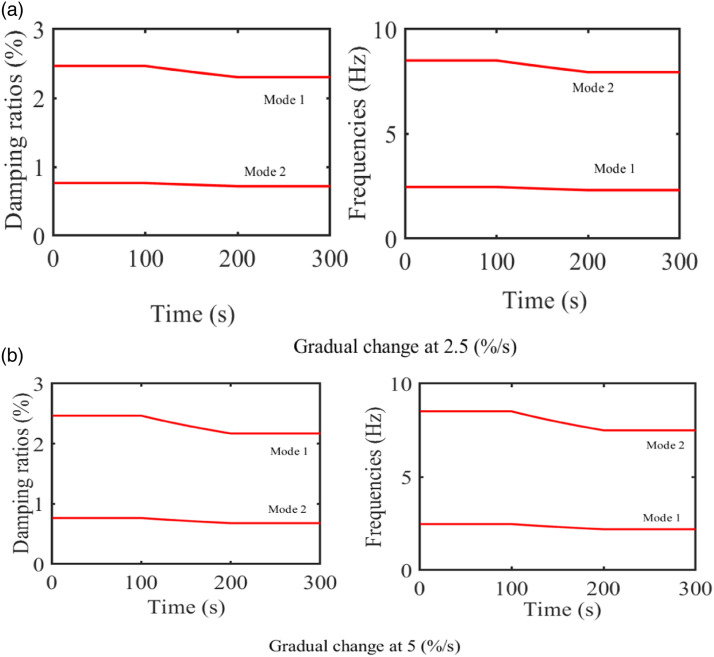
Modal parameters of the system.

The system’s varying modal parameters are simulated in Figure 5 on a theoretical basis for the two different rates, 2.5 (%/s) and 5 (%/s). At the masse’s change rate 2.5 (%/s), the first natural frequency varies within the range of (2.32–2.47) (Hz), and the second varies within the range of (7.95–8.51) (Hz). At the masse’s change rate 5 (%/s), the two natural frequencies vary within the range of (2.19–2.47) (Hz) and (7.49–8.51) (Hz), respectively.

**Figure 6.**
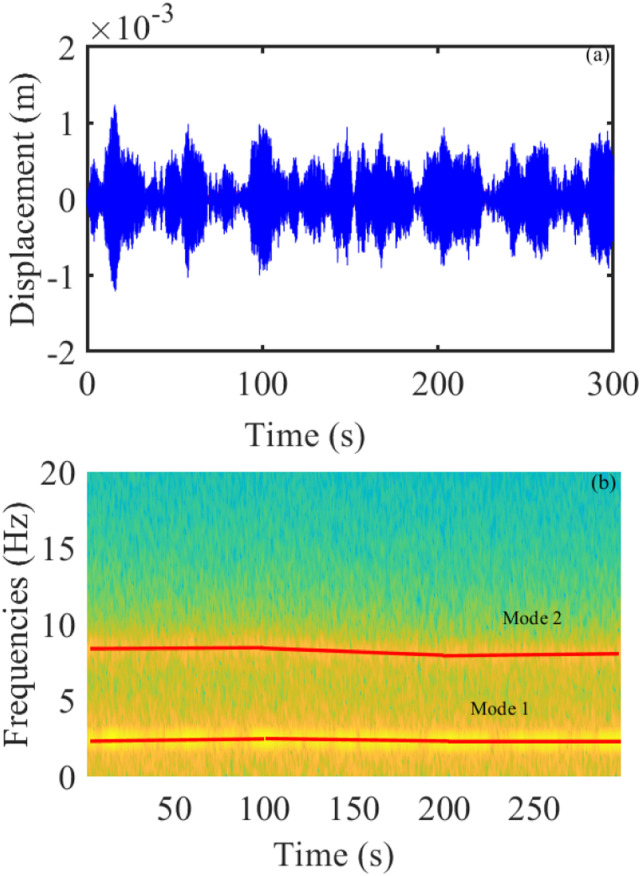
(a) Realization of the non-stationary vibration displacement and (b) Short-time Fourier transform of the signal with the gradual change at 2.5 (%/s).

**Figure 7.**
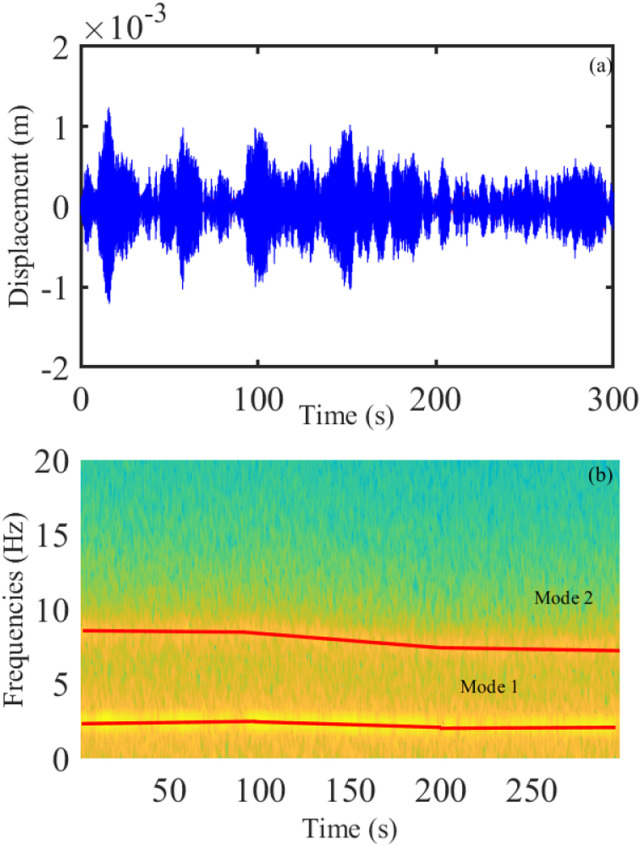
(a) Realization of the non-stationary vibration displacement and (b) short-time Fourier transform of the signal with the gradual change at 5 (%/s).

The displacement responses of the system under a particular excitation were obtained through the ODE45 method in MATLAB using a fixed-integration step and recorded at a sampling frequency of 100 (Hz). At the mass change rate 2.5 (%/s), Figure 6(a) plots the non-stationary vibration displacement signal. The signal’s spectrogram under a random excitation is presented Figure 6(b). Figure 7(a) and (b) show the non-stationary vibration displacement signal and the signal’s spectrogram under a random excitation at the mass change rate 5 (%/s).

The proposed method uses the AR model to identify the mechanical system’s modal parameters in each data segment. The model’s parameters at the next segment are updated using the results of previous segments. The length of each window is sufficient to contain the modal parameters, and the length must be at least four times the highest period in order to track the changes of all modes. The model order selection procedure is based on the minimum description length (MDL) (Bui et al., 2022).

**Figure 8.**
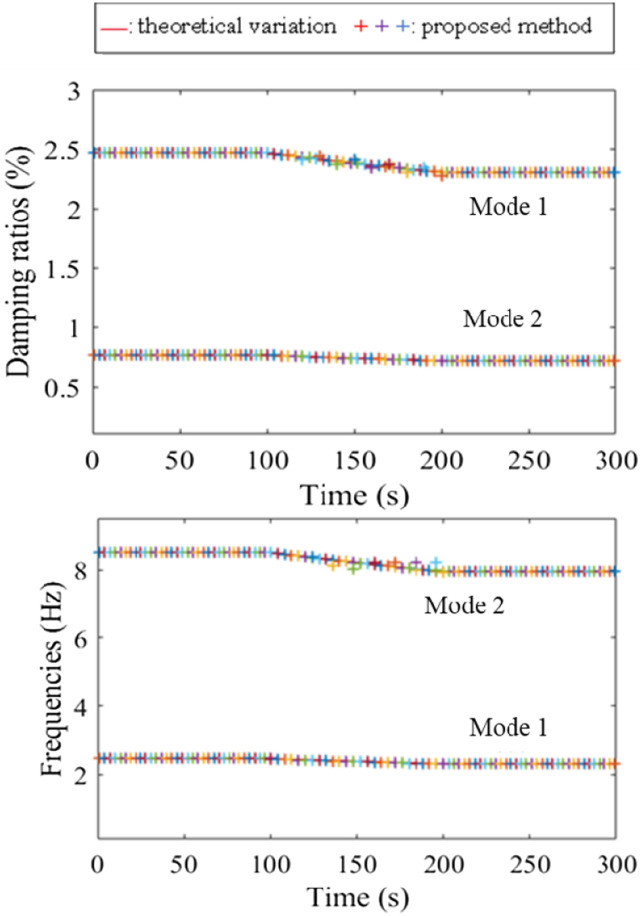
Modal parameters of the system with the gradual change at 2.5 (%/s) using the proposed method.

**Figure 9.**
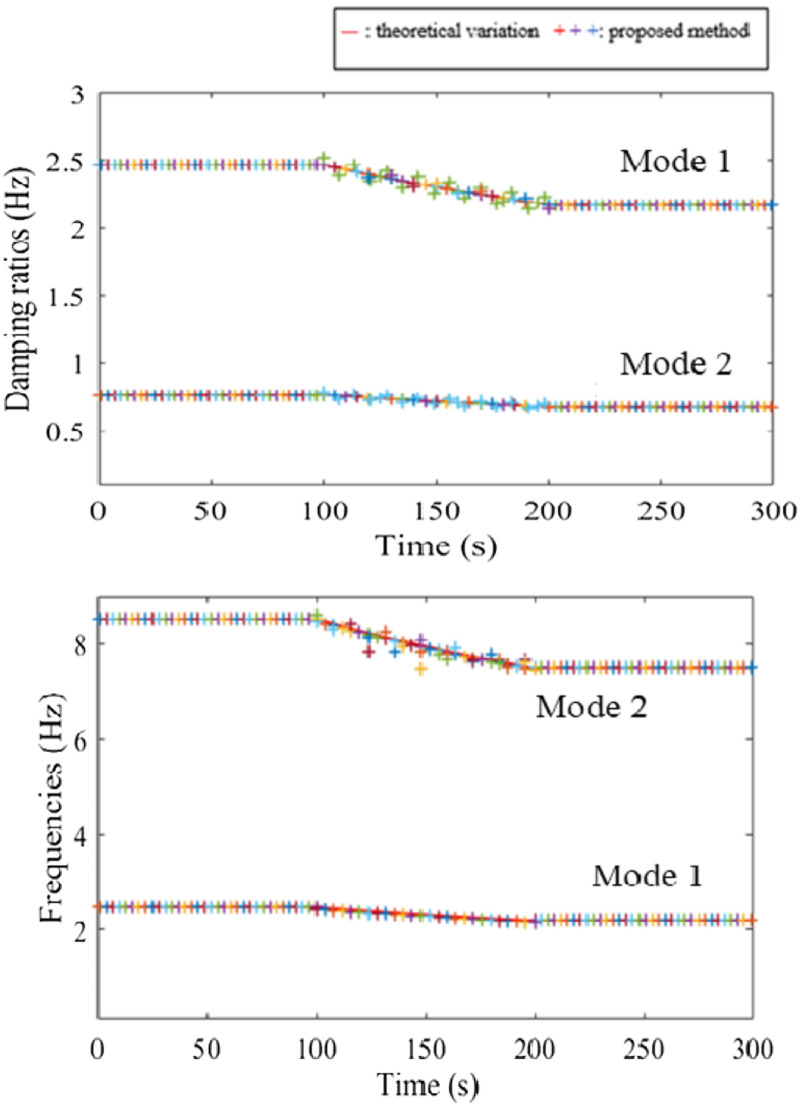
Modal parameters of the system with the gradual change at 5 (%/s) using the proposed method.

Figure 8 presents the identification of the modal parameters with the proposed method at the mass change rate 2.5 (%/s). Obviously, the natural frequencies are accurately determined and tracked. The results of damping ratios for the first and second modes are also in very good agreement with the calculated values. The proposed method performs better in terms of accuracy resolution than STFT. It can be observed from these figures that the proposed method matches well with theoretical variations. At the higher change rate of the masses, the modal parameters of the system using the proposed method are shown in Figure 9.

Table 1 presents a comparison of the computation time for the modal parameter diagrams of the proposed method with the recently published updated method by Schur complement (Bui et al., 2022). Generally, the identified results by the proposed method match with the simulated results presented in Figure 5. It can be seen that the first mode is still accurately identified and tracked. However, the second mode is largely dispersed, especially its damping ratio, which is known to associate with a greater uncertainty (Vu et al., 2011a). Conservatively, the proposed method can identify and monitor the mass change rate at 5 (%/s). This change rate is quite high for real systems. As can be seen from Table 1, the proposed method performs better than the Schur method.

**Table 1.** Comparison of the computational time of two methods: method by Schur complement (Bui et al., 2022) and proposed method for modal parameter diagram (data from two-channels).

	Method by Schur complement (Bui et al., 2022)		Proposed method	
Mass change	2.5 (%/s)	5 (%/s)	2.5 (%/s)	5 (%/s)
Time (s)	114	114	103	103

### 5.2. Experimental data

**Figure 10.**
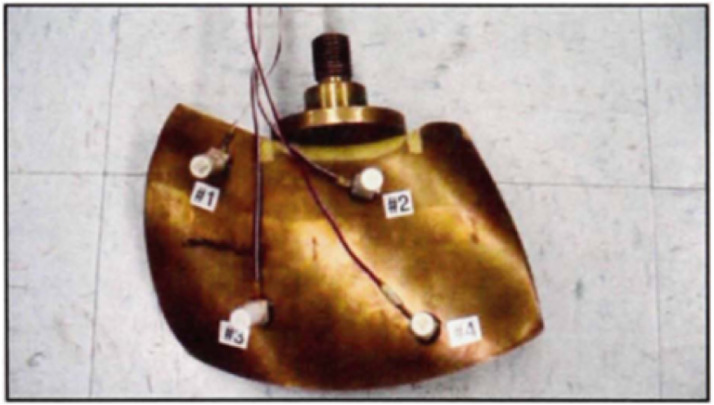
Blade with four accelerometers.

In this section, the proposed method is applied to a hydraulic turbine blade to monitor the modal parameters. The turbine blade is made of bronze alloy “M”-C92300 corresponding to the standard designation 87Cu-8Sn-IPb-4Zn.F. Figure 10 shows the configuration of the test in which four accelerometers have been mounted to record the accelerations of this blade.

#### 5.2.1. The turbine blade in air

**Figure 11.**
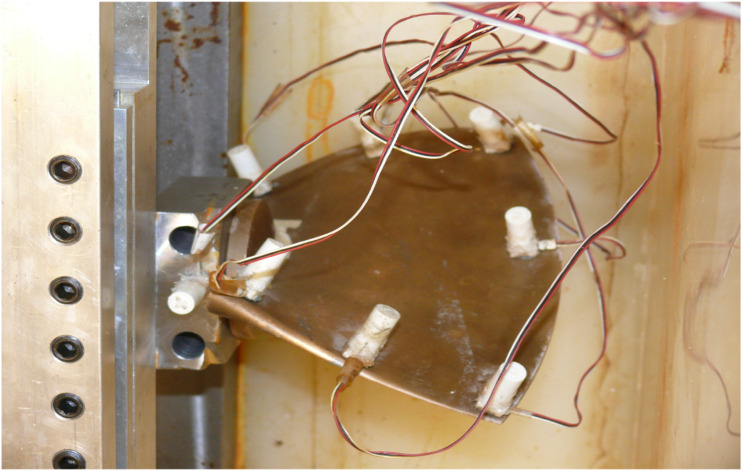
Modal test of the blade in the air.

The test was carried out using an LMS system. A PCB impact hammer with a sampling frequency of 6400 (Hz) acted on the structure in the static test (Figure 11). The natural frequencies of this blade shown in Table 2 are obtained by different methods: Ansys, the power spectral density (PSD) of responses in MATLAB, short-time autoregressive model (STAR) (Vu et al., 2011a), method by Schur complement (Bui et al., 2022), and the proposed method. As seen, the proposed method reveals excellent matching results with previous identification methods.

**Table 2.** Modal identification of the blade in the air (Hz).

	Mode 1	Mode 2	Mode 3	Mode 4	Mode 5	Mode 6
Ansys	Non-identify	332.9	643.16	896.9	963.6	1157.6
PSD in MATLAB	204.7	359.4	582.8	843.8	1050	1295.0
STAR (Vu et al., 2011a)	208.0	368.0	576.0	848.0	1040	1294.5
Method by Schur complement (Bui et al., 2022)	204.0	357.2	579.4	845.9	1047	1293.0
Proposed method	204.4	360.3	582.5	846.4	1055	1297.0

**Figure 12.**
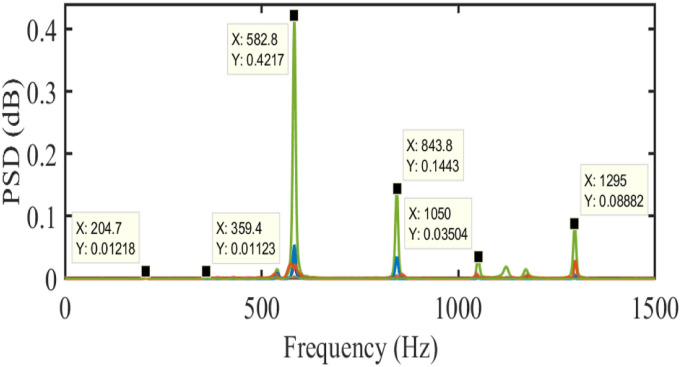
Spectra of the blade in the air by spectrogram functions in MATLAB.

**Figure 13.**
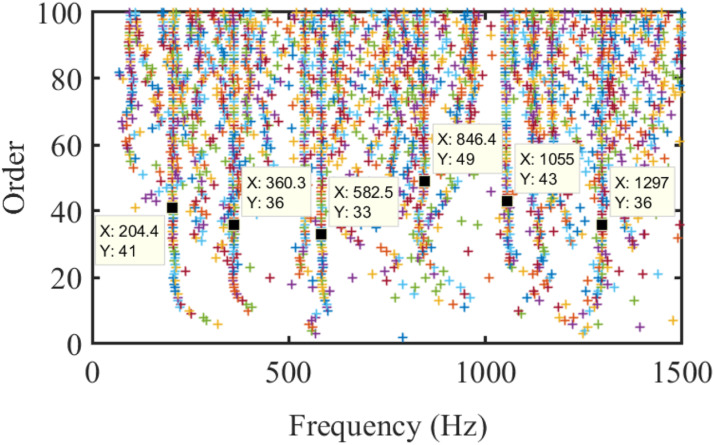
Order-updating stabilization diagram of the blade in the air using the proposed method.

The first frequency (204.7 Hz) was not identified using the finite element method because it was the mounting-structure mode. The system’s PSD plot obtained from the spectrogram function in MATLAB is presented in Figure 12. The stabilization diagram obtained from the proposed method is shown in Figure 13. As shown in Table 2, the identifications of frequencies by the proposed method are pretty accurate for all modal parameters. The proposed method with order updating was applied to the systems to extract the natural frequencies.

#### 5.2.2. Turbine blade raised from water

##### 5.2.2.1. Experiment setup

A hydraulic test bench was constructed in the research laboratory to evaluate fluid-structure interactions. When the blade was placed inside a perforated tank, the vibration of the blade in air and water could be measured. The valves and various outlet nozzles are used to control the flow speed. The flow rates and therefore the depth of the blade in the water can be adjusted according to the desired flow velocities. A fastening system is a multi-tasking tool for mounting a blade in variable boundary conditions.

**Figure 14.**
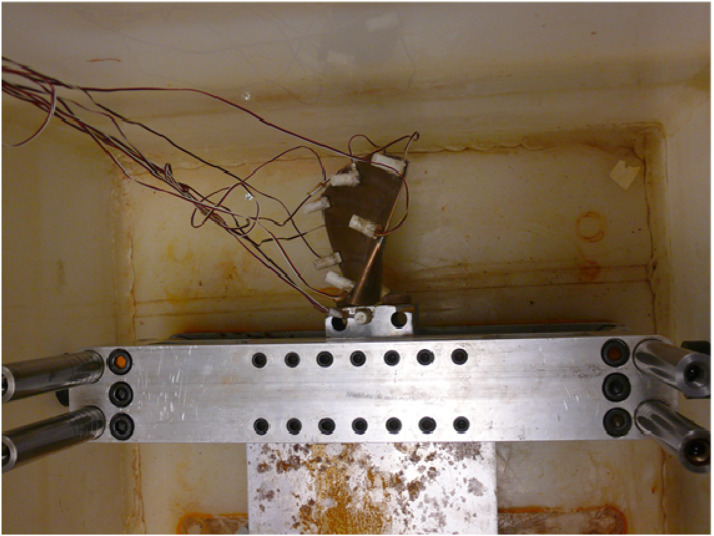
Modal test of the blade in water.

The PCB330A sensors are utilized for vibration measurements. The Vishay System 6000 was used to record pressure sensors during different tests. The data is exported and saved in different formats, such as.xls and .txt, by the software “Strain Smart” designed to work with the Vishay acquisition box. A PCB impact hammer is acted on the structure tests. The hammer is equipped with a steel extension to hit the plate at different depths. The configuration of the experiment is depicted in Figure 14. The blade is submerged at different depths, and the depth/length ratio varies from 0.4 (totally submerged) to 0 (in air).

**Figure 15.**
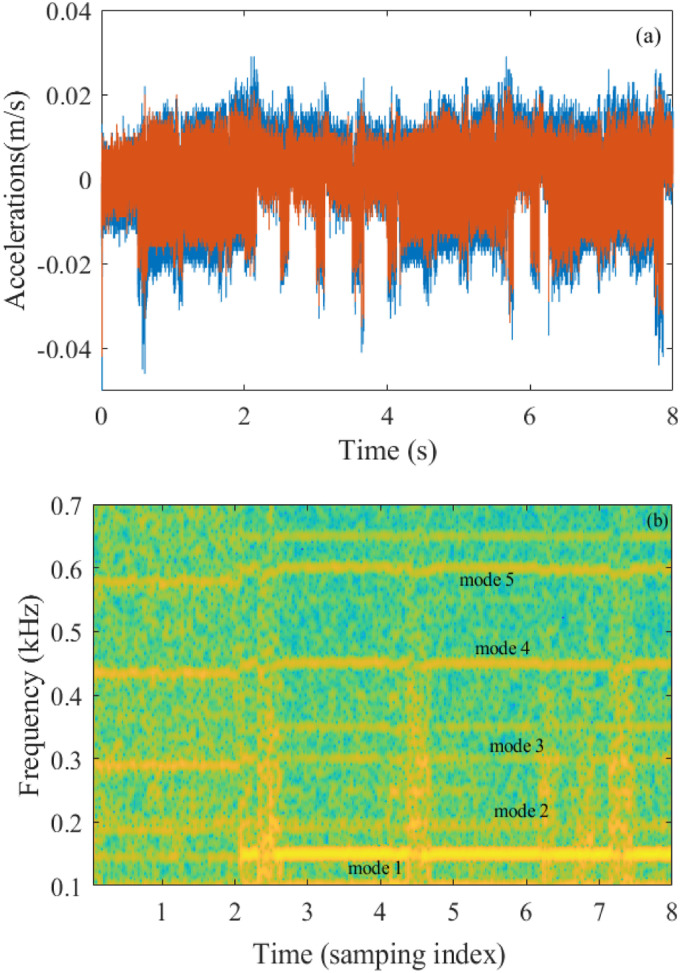
(a) Response to the blade's acceleration and (b) short-time Fourier transform of the signal.

The natural frequencies of the blade change with respect to the submerged depth due to the effect of the fluid. Before the blade rises, its modal parameters were determined by analytical and experimental methods at different depth length ratios (D/L). The result is shown in Table 3. The temporal response data at sampling frequency 8192 (Hz) are depicted in Figure 15(a) and (b) plots the short-time Fourier transform of the signals.

**Table 3.** Modal identification of submerged blade by impact test.

Mode	Frequencies (Hz) of submerged condition (depth/blade length)			
	0.4 (totally submerged)	0.2	0.1	0 (totally in air)
1st	160.8	166.85	170.8	Non-identify
2nd	288.1	288.1	290	332.9
3rd	352.7	354	358	643.2
4th	496.9	498	500	896.9
5th	640.5	642.8	645	963.6

The modal signal-to-noise ratio (Vu et al., 2007) is utilized to separate spurious and real modes. The smallest frequency is then obtained for the next window computation. In this section, the proposed method was applied to track modal parameters for the submerged blade. The length of each data segment was chosen to track all the modes of the signals. The size length of the sliding window varies and is chosen to be at least 4 times that of the longest natural period of the previous block (Bui et al., 2022).

Before the blade rises, its modal parameters was determined by analytical and experimental methods at different depth length ratios (D/L). The result is shown in Table 3.

**Figure 16.**
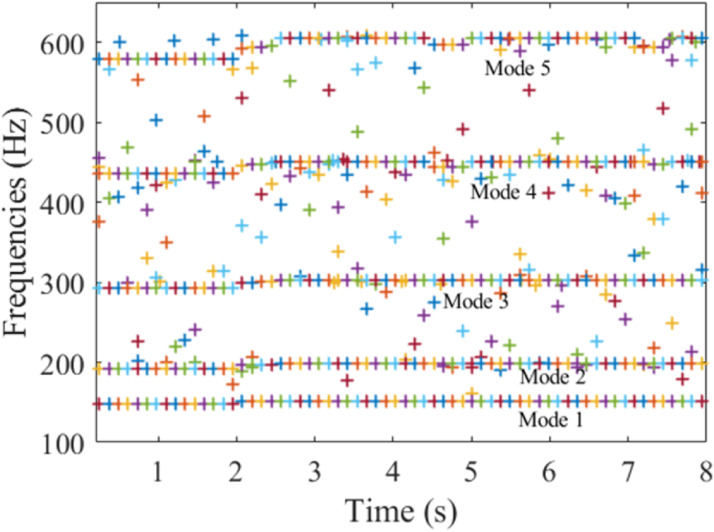
Natural frequencies of the blade using the proposed method.

In this section, the proposed method was applied to the measured signals. The length of each data segment was chosen to track all the modes of the signals. In each window, the minimum description length (MDL) was used to obtain the order of the models. The system's natural frequencies were extracted using the proposed method as shown in Figure 16.

Compared with the modal testing in Table 3 and STTF of the signals shown in Figure 15, the identifications of frequencies (147–151.6, 191.2–199, 292.2–303, 435.3–451.4, 579.6–605.2) (Hz) are quite accurate for all modal parameters. Figure 16 shows that the natural frequencies increase slightly when the blade rises from the water to the surface under turbulence. This increase is relevant to the depth ratio of the blade submerged in water. This result agrees well with the conclusion in other authors (Vu et al., 2007).

During the rise-up, the blade is in a moving condition, and the boundary condition is affected by the movement. The clamp and the fixing systems are also in vibration. These lead to a little discrepancy to results in Table 3 which are from the modal testing (static), Figure 16 is dynamic turbulent testing, so some other effects, such as the boundary condition, change of mass, and stiffness, can explain the discrepancy. However, the two representations agree on the frequencies and the variation of the frequencies.

## 7. Conclusion

In this work, a new method of the modal parameter identification and monitoring using a vector autoregressive model has been introduced for non-stationary vibrations. The technique uses SVD and a short-time sliding window as autoregressive models to extract the model parameters. The method aims to reduce the computational complexity for the online slow-varying monitoring of non-stationary cases by updating the SVD to estimate AR model parameters through the order and time of the previous computational window. The proposed method was validated first through a numerical simulation of a mechanical system at the different rates of masses and then through experiments on a submerged hydraulic turbine blade. Results show that the proposed method is a powerful technique for analysis and monitoring the modal parameters in non-stationary vibration systems under a reasonable varying rate at 5% per second. Natural frequencies can be accurately obtained and monitored. The identification and monitoring of damping ratios are sufficiently convincing.
